# Supplementary material for: Mental health issues of children and young people displaced by conflict: A scoping review
Source: PLOS Ment Health. 2024 Nov 20;1(6):e0000076. doi: 10.1371/journal.pmen.0000076 (PMC12798309; doi:10.1371/journal.pmen.0000076)
Supplement: Attachment — Submitted filename: Response to Reviewer (PLOS-MH 1).docx [file pmen.0000076.s002.docx]

**Response to Reviewers**

We sincerely appreciate the editorial team and the reviewers for taking the time to review this manuscript and for the valuable feedback we have received. Here is our point-by-point response to the reviewer comments.

**Reviewer #1:** 
Kindly fix the sentence framing in the introduction part, kindly explain Rayyan website as well as include adjustment disorder if you have seen in literature.

**Response Point By Point**

- The framing of the sentence in the introduction part has be reviewed to enhance comprehension as can be seen in the paragraphs 1-3
- A concise explanation of Rayyan website has been provided under data sources as suggested.
- Unfortunately, the review did not turn up relevant literature on adjustment disorders in the study population hence it was not included.

**Reviewer #2:** Data source - explicitly described in the paper well written paper- structured and easy to read; the scope of this study was strictly defined as a scoping review which confined the parameters to strict inclusion/exclusion criteria. The papers cited/ references suitable relevant

Additional comments to editor:

I would suggest that the authors use the UNHCR data on forced displacement closest to the time of study or to cited reference which is 2023 - should be at least 2022 here: <https://www.unhcr.org/sites/default/files/2023-06/global-trends-report-2022.pdf> add some additional context relevant to this study- children make up 40% of all forcibly displace people. was there a reason why gender was not included in the Data Charting? linked the words "female sex" used in the text- need some clarification as - there is evidence of rapes, gender based violence, other sexual exploitations of displaced people including very young girls and sometimes boys

**Response Point By Point**

- The suggestion that the updated UNHCR data on forced displacement closest to the time has been adopted using data from the URL provided: <https://www.unhcr.org/sites/default/files/2023-06/global-trends-report-2022.pdf>
- “children make up 40% of all forcibly displaced people” This suggestion is evidenced based and has been fully incorporated and cited appropriately as suggested.
- Yes, gender was not explicitly included in the data charting because the primary objective of the study was to focus on mental health issues across younger children, older adolescents, and young people as a sub population, identify factors that affect their mental health and appraise the effectiveness of interventions. However, the study's results still offered valuable insights into gender differences, identifying specific risks and their distinct impacts on mental health outcomes, ensuring that both male and female experiences were adequately captured and discussed in the final analysis.

**Reviewer #3**: Thank you for the opportunity to review this article on an important topic of mental health and mental health services of children and adolescents impacted by forced displacement.
Unfortunately, and based on my careful review of the abstract, background and methods section, I am not able to recommend this article for publication. There are significant issues with the paper that require attention.

Irrespective of the opinion provided by the reviewer, we still want to sincerely appreciate the reviewer for taking time to review the manuscript. Here is our point by point reponse to the remarks presented

The background section is not well cited and does not adequately engage with the empirical literature on this topic which is quite extensive. More detail and specificity on what is known and gaps that remain are needed to enhance the quality of the background section. More importantly, there are a number of different reviews of mental health of children impacted by war, conflict and forced displacement which have already been conducted and published. There needs to be a thorough discussion of this existing literature and clear distinction of what is known from these reviews and what gaps remains which this review seeks to address.
Overall, the quality of writing needs improvement with overall copyediting needed.
The methods section overall is lacks key details (some of which I have elaborated below) related to guidelines that were used to conduct the study, search terms and how they were chosen, clarification of inclusion criteria and how selected records were reviewed and appraised. Based on the description, it is not clear if the data extraction tool was develop a priori (a requirement for review) or post-review.
There are currently no details on the analytic methods employed to analyze the data. I have included some additional detailed comments below to elaborate on my summary here and which I hope may be helpful to the authors as they continue to work on their manuscript.
The background literature section is not detailed enough in terms of summarizing the state of the existing literature on forcibly displaced populations, what key findings have been identified? With what populations? Etc. The background literature is also not well-cited in a number of places. There have been many different reviews of the literature that need to be referenced and within which, the current review should be situated. The reviews that appear to be cited seem specific to the African context.
Overall, I find the background section to be lacking rigor in terms of really teasing apart the key issues and needing much more specificity.
Much more engagement with the existing literature on mental health concerns, interventions and services of children and adolescents impacted by forced displacement is needed, particularly engagement with existing reviews which have been conducted. Given that numerous reviews on this topic have already been conducted, what gap is this scoping review seeking to address? The particular rationale for this paper is not clear in the existing manuscript.
Methods
Authors need to specify which specific review guidelines were followed to conduct this review. Please provide a working definition for the scoping review undertaken.
Included a detailed list of inclusion criteria.
Explain more detail about how the relevant databases were selected. Please include details on the study protocol that was developed and if the protocol was registered or is accessible.
Indicate when the searches were conducted and by whom.
Include an appendix with full search and mesh terms for the various databases. Some of the keywords appear problematic as they are included as plurals which may have limited the authors potential to identify relevant studies.
More detail on the title/abstract review and full text review are needed. Who conducted those reviews, how were conclusions reached, why were articles excluded?
There are no details on the methods of analysis used to examine and synthesize

**Response Point By Point**

- Our study clearly cites several studies that have collected empirical data on the mental health of children and young people impacted by war, conflict, and forced displacement, analyzing their findings and highlighting gaps in the existing research. The background section has also been updated to include more empirical literature, providing evidence based on real-world data.
- We appreciate the comment regarding the need to distinguish our review from previous ones. This distinction was clearly and explicitly addressed in the "Rationale and Knowledge Gap" section of the manuscript. While several studies exist on the physical, sexual, and reproductive health effects of displacement on the general population, women, and children, there is a noticeable gap in research focused specifically on the mental health of displaced children, particularly adolescents and young people. Therefore, the aim of our study was to identify all existing research on the mental health needs of displaced children and youth, addressing this gap and highlighting future research needs for policymakers and humanitarian agencies to design programs that effectively address the mental health challenges of this population.
- We acknowledge the need for overall copyediting to improve the quality of writing and we have ensured a thorough proofreading the revised manuscript to enhance clarity, grammar, and overall coherence, addressing the current issues with the writing quality.
- Our study adhered strictly to the scoping review guidelines and provided full details on process followed (PRISMA). The inclusion and exclusion criteria for studies in this scoping review were clearly outlined as the first item in the methodology section, elaborating on the search terms, the rationale behind their selection, and the protocols that guided the process. Furthermore, explanation of the use of the Rayyan website for screening and organizing studies has now been fully explained to provide better context and enhance transparency as earlier suggested.
- We appreciate the need for clarity on whether the data extraction tool was developed a priori. This has been explicitly addressed in the revised methodology, confirming that the tool was developed prior to data extraction.
- Studies have shown that Africa, particularly Sub-Saharan Africa, and many developing countries bear a significant percentage of conflict and displacement among young people. Sub-Saharan Africa alone hosts 46 percent of the world's internally displaced persons (IDPs), which explains why much of the empirical literature has an African context.

**References**

Ndegwa DG. Contemporary Forced Migration in Africa. InRoutledge Handbook of Contemporary African Migration 2023 Sep 1 (pp. 182-196). Routledge.

Musa A, Aide A, Bayero AA, Shuaibu M. Globalization, Conflict and Security in Africa. Kashere Journal of Politics and International Relations. 2023 Sep 22;1(1).

<https://www.unhcr.org/sites/default/files/2023-06/global-trends-report-2022.pdf>

https://www.internal-displacement.org/global-report/grid2024/

Once again, we wish to appreciate the editorial team and the reviewers for taking their time to review our manuscript.

**Authors**
